# Permanent pacemaker implantation with high pacing rate for treating refractory right heart failure: a case report

**DOI:** 10.1093/ehjcr/ytaf618

**Published:** 2025-11-29

**Authors:** Yanxing Fang, Wenzhi Pan, Daxin Zhou, Junbo Ge

**Affiliations:** Department of Cardiology, Zhongshan Hospital, Fudan University, No. 180, Fenglin Road, Shanghai 200032, China; National Clinical Research Center for Interventional Medicine, Zhongshan Hospital, Fudan University, No. 180, Fenglin Road, Shanghai 200032, China; Department of Cardiology, Zhongshan Hospital, Fudan University, No. 180, Fenglin Road, Shanghai 200032, China; National Clinical Research Center for Interventional Medicine, Zhongshan Hospital, Fudan University, No. 180, Fenglin Road, Shanghai 200032, China; Department of Cardiology, Zhongshan Hospital, Fudan University, No. 180, Fenglin Road, Shanghai 200032, China; National Clinical Research Center for Interventional Medicine, Zhongshan Hospital, Fudan University, No. 180, Fenglin Road, Shanghai 200032, China; Department of Cardiology, Zhongshan Hospital, Fudan University, No. 180, Fenglin Road, Shanghai 200032, China; National Clinical Research Center for Interventional Medicine, Zhongshan Hospital, Fudan University, No. 180, Fenglin Road, Shanghai 200032, China

**Keywords:** Permanent pacemaker implantation, Pacing rate, Right-sided heart failure, Haemodynamics, Quality of life, Case report

## Abstract

**Background:**

Current guidelines recommend that patients with systolic heart failure should keep their resting heart rate (HR) at a relatively low level. However, the optimal HR level for patients with right-sided heart failure (RHF) remains unclear.

**Case summary:**

A 43-year-old male diagnosed with desmin-related cardiomyopathy 3 years ago has been repeatedly hospitalized over the past 5 months due to fatigue and severe oedema, with suboptimal control despite high-dose diuretic medication. This patient, with an average HR of 55–60 b.p.m., was implanted with a pacemaker adjusted at 90 b.p.m. on this admission. Right heart catheterization revealed immediate improvements in haemodynamics, including increased cardiac output and decreased vena cava pressure and right atrial pressure. At the 12-month follow-up, the patient was classified as New York Heart Association Class I and did not experience recurrence of oedema, even while taking low-dose diuretics (torasemide, 5 mg once daily, and spironolactone, 20 mg once daily).

**Discussion:**

A previous study has shown that personalized acceleration of pacing rate in patients with heart failure with preserved ejection fraction can effectively improve quality of life. In addition, patients who undergo Fontan surgery can remain asymptomatic for decades, suggesting that left ventricular pump function may be sufficient to sustain both systemic and pulmonary circulation. Therefore, increasing HR can increase cardiac output, promote forward flow from the right atrium and vena cava into the pulmonary circulation, and reduce venous and right atrium pressures in patients with RHF and normal left-sided heart function.

Learning points

- Current guidelines do not specify the optimal heart rate (HR) level for patients with right-sided heart failure (RHF).
- Increasing HR could improve haemodynamics and quality of life for patients with refractory RHF and normal left ventricular ejection fraction.

## Introduction

Previous studies and consensus suggested that the heart rate (HR) of patients with systolic heart failure should be controlled at a relatively low level (50–60 b.p.m.).^1–4^ However, in patients with heart failure with preserved ejection fraction, HR-lowering agents such as ivabradine or β-blockers were associated with adverse clinical outcomes.^5,6^ Furthermore, the appropriate level of HR in patients with right-sided heart failure (RHF) remains unexplored.

Patients who undergo Fontan surgery can remain asymptomatic for decades, suggesting that left ventricular pump function could effectively sustain both systemic and pulmonary circulation.^7^ Therefore, we hypothesized that for patients with RHF who have normal left-sided cardiac function, increasing HR could enhance cardiac output (CO), reduce venous and right atrium pressures, and improve congestion symptoms. We previously prospectively recruited 10 patients with RHF who had already received pacemaker implants. After increasing their pacing rate to 90 b.p.m., we observed significant improvements in both haemodynamic parameters measured by right heart catheterization and quality-of-life scores at the 3-month follow-up.^8^ In this case, we implanted a permanent pacemaker and adjusted the pacing rate to 90 b.p.m. for a patient with refractory RHF to validate this hypothesis.

## Summary figure

**Figure:**
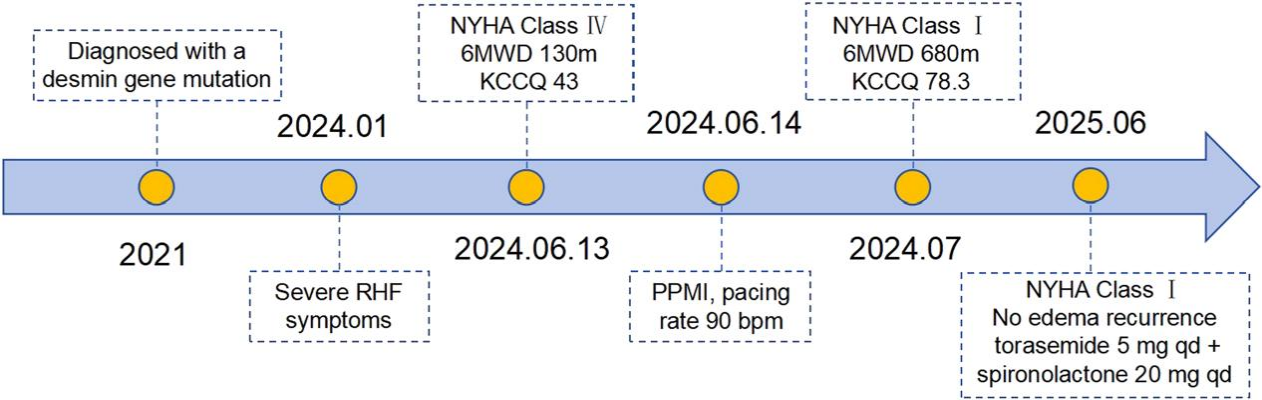


## Case presentation

**Figure 1:**
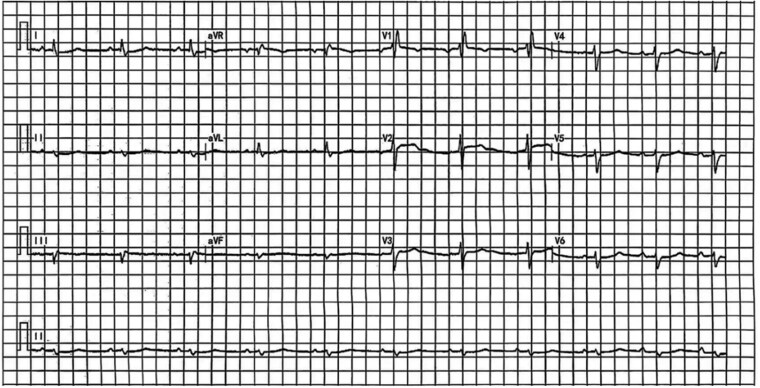
Electrocardiogram before pacemaker implantation. Electrocardiogram revealed a heart rate of 62 b.p.m., sinus rhythm, complete right bundle branch block, and first-degree atrioventricular block.

A 43-year-old man was referred to our hospital with chief complaints of shortness of breath, bloating, and severe oedema for 5 months, and worsening for 2 weeks [New York Heart Association (NYHA) Class Ⅳ]. On admission, his liver and kidney function, thyroid function, and routine blood test results were normal. The patient's N-terminal pro-brain natriuretic peptide (NT-proBNP) level was measured at 786.2 pg/mL. The patient had a self-measured resting HR between 55 and 60 b.p.m. The current admission electrocardiogram revealed an HR of 62 b.p.m., sinus rhythm, complete right bundle branch block, and first-degree atrioventricular block (*Figure 1*). Echocardiography revealed a left ventricular ejection fraction (LVEF) of 69%, left atrial diameter of 47 mm, left ventricular end-diastolic diameter of 42 mm, left ventricular end-systolic diameter of 26 mm, and pulmonary artery systolic pressure of 28 mmHg. The right atrium was enlarged with a supero-inferior dimension of 71 mm, and the right ventricular end-diastolic diameter was also increased, measuring 43 mm at the basal level. Furthermore, right ventricular wall systolic motion was diminished, with a tricuspid annular plane systolic excursion (TAPSE) of 9 mm, and moderate tricuspid regurgitation was present (*Table 1*). Functional assessments demonstrated a 6-min walk distance (6MWD) of 130 m and a Kansas City Cardiomyopathy Questionnaire (KCCQ) score of 43 out of 100. Over the previous 2 months, he had developed progressive fatigue and difficulty walking. Despite the use of large amounts of diuretics (torasemide, 40 mg b.i.d.; spironolactone, 20 mg b.i.d.; and tolvaptan, 15 mg q.d.), his symptoms worsened, and urine output was <500 mL/day. In addition, he had severe lower limb oedema that affected the thighs and testicles, and he was unable to climb stairs. Ultrasound revealed a large amount of ascites and pleural effusion, which was drained and treated with torasemide and dobutamine. In 2018, the patient underwent radiofrequency ablation for atrial flutter at our hospital. In 2021, he was diagnosed with a desmin (DES) gene mutation, suggesting DES-related cardiomyopathy. The patient had previously undergone chest computed tomography, computed tomography pulmonary angiography, and echocardiography, excluding lung parenchyma disease, chronic thrombo-embolic pulmonary hypertension, and left-to-right shunts.

**Table 1:** Echocardiography data at the baseline, 3-month, and 12-month follow-ups

	Baseline	3-month	12-month
LVEDD (mm)	42	43	37
LVESD (mm)	26	27	22
RVEDD	43	43	41
PASP (mmHg)	28	28	30
TAPSE (mm)	9	12	12
LAD (mm)	47	44	39
RAD (mm)	71	66	54
LVEF (%)	69	67	70
Degree of MR	0	0	0
Degree of TR	2	1	2

LVEDD, left ventricular end-diastolic diameter; LVESD, left ventricular end-systolic diameter; RVEDD, right ventricular end-diastolic diameter; PASP, pulmonary artery systolic pressure; TAPSE, tricuspid annular plane systolic excursion; LAD, left atrial diameter; RAD, right atrial diameter; LVEF, left ventricular ejection fraction; MR, mitral regurgitation; TR, tricuspid regurgitation.

**Figure 2:**
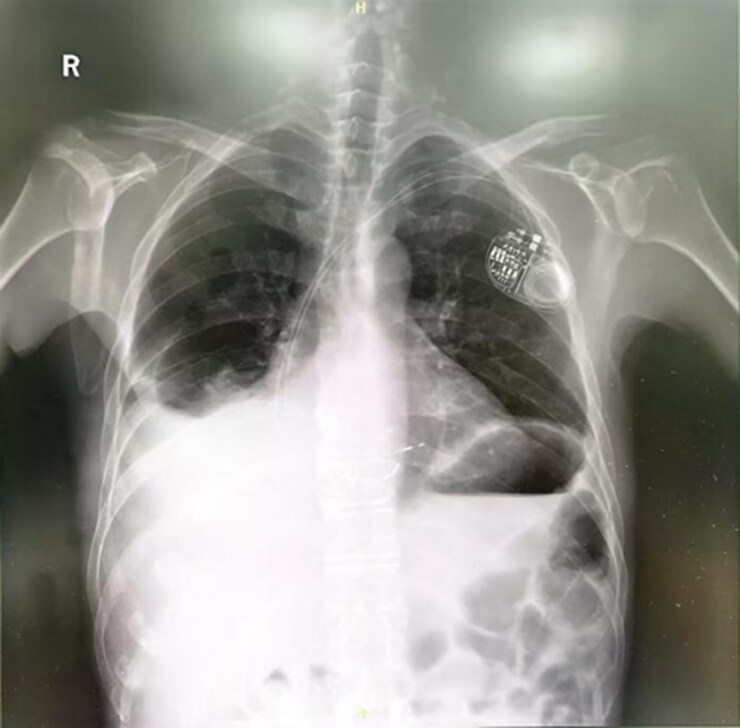
Chest X-ray after pacemaker implantation. The electrode leads were positioned in the right atrial appendage and the interventricular septum.

**Figure 3:**
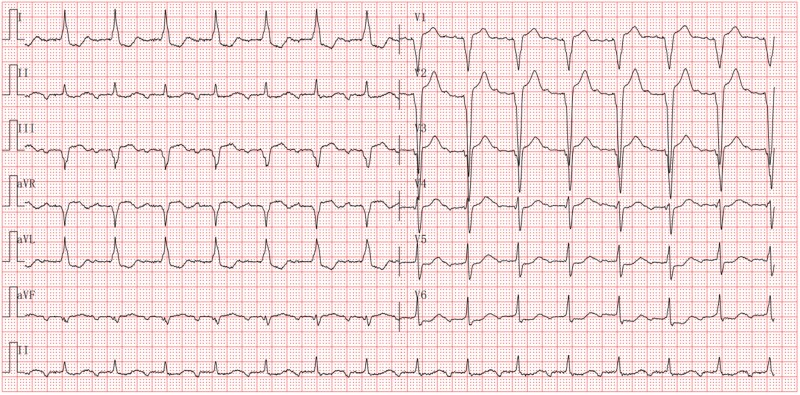
Electrocardiogram after pacemaker implantation. The electrocardiogram demonstrates DDD pacing mode, with a heart rate of 90 b.p.m.

We believe that in patients with right heart failure and preserved left ventricular function, increasing the HR can enhance CO and reduce atrial pressure, thereby alleviating congestive symptoms. Consequently, after thorough discussion and obtaining the patient's informed consent, we proceeded with the pacemaker implantation and subsequent rate acceleration based on compassionate use considerations. As shown in *Figure 1*, the patient had a preoperative PR interval of 220 ms, indicating first-degree atrioventricular block. We implanted a dual-chamber pacemaker with a ventricular electrode positioned in the left bundle branch area (*Figure 2*), and initially attempted AAI pacing mode with a paced atrioventricular interval of 280 ms, but atrioventricular conduction failure occurred. Considering potential issues with the patient's conduction system, we ultimately programmed the sensed atrioventricular interval to 210 ms and the paced atrioventricular interval to 240 ms, with the pacing mode set to DDD. However, the electrocardiogram showed fusion QRS complexes after the procedure, indicating that the left bundle branch pacing was not achieved (*Figure 3*). The HR was adjusted to 90 b.p.m. during the day and 70 b.p.m. at night.

Before the procedure, right heart catheterization was performed under spontaneous rhythm. After pacemaker implantation, right heart catheterization was repeated immediately, and the haemodynamics improved significantly (*Table 2*). The CO increased from 2.7 to 3.9 L/min, while the vena cava pressure and right atrial pressure also significantly decreased. After the pacemaker implantation, the patient reported a stronger pulse. During maintenance with low-dose diuretics (torasemide, 10 mg q.d., and spironolactone, 20 mg q.d.), the daily urine output of this patient was >2000 mL. The oedema regressed, and the shortness of breath was relieved gradually. This patient was discharged 5 days after the procedure with an NT-proBNP level of 458.6 pg/mL.

**Table 2:** Haemodynamics assessed by right heart catheterization before and after pacemaker implantation

	Baseline	HR at 90 b.p.m.
SVC (mmHg)	18/13/16	8/4/6
IVC (mmHg)	20/11/15	9/4/6
RA (mmHg)	19/10/15	9/5/7
RV (mmHg)	28/10	27/3
PA (mmHg)	28/8/15	26/8/14
PCWP (mmHg)	15/7/10	14/7/10
CO (L/min)	2.7	3.9

SVC, superior vena cava; IVC, inferior vena cava; RA, right atrium; RV, right ventricle; PA, pulmonary artery; PCWP, pulmonary capillary wedge pressure; CO: cardiac output.

SVC, IVC, RA, PA, and PCWP pressures are presented as systolic, diastolic, and end-diastolic values. Right ventricular pressures are presented as systolic and diastolic values.

Functional assessments at the 1-month follow-up demonstrated a 6MWD of 680 m and a KCCQ score of 78.3. Echocardiography data at the 3- and 12-month follow-ups are presented in *Table 1*. The right atrial diameter decreased from 71 to 54 mm while left heart size decreased, and the LVEF was maintained. The patient was classified as NYHA Class I and did not experience recurrence of oedema at the 12-month follow-up, even while taking low-dose diuretics (torasemide, 5 mg q.d., and spironolactone, 20 mg q.d.). At the 1-year follow-up, the right ventricular pacing percentage was 99.7%, and the right atrial pacing percentage was 99%. In addition, the patient's NT-proBNP level at the 6-month follow-up was measured at 312.4 pg/mL.

## Discussion

In this case, we report a patient with RHF who was refractory to high-dose diuretics. Although the patient's electrocardiogram did not show sufficient justification for permanent pacemaker implantation, after pacemaker implantation and increasing his daytime pacing rate to 90 b.p.m., right heart catheterization demonstrated immediate haemodynamic improvement: CO increased, and vena cava pressures decreased. Remarkably, during the 12-month follow-up after discharge, the patient has maintained NYHA Class I functional status with only low-dose diuretic therapy. His quality of life and oedema have significantly improved, suggesting that increasing HR may be a simple and effective approach to address refractory RHF in the long term.

Heart rate was an important control target for cardiovascular disease, especially for left ventricular systolic heart failure.^1,2^ Current guidelines suggest HR should be controlled to 50–60 b.p.m. in these patients.^3,4^ However, the optimal HR level for patients with RHF remains unclear. A previous study has shown that personalized acceleration of pacing rate in patients with heart failure with preserved ejection fraction can effectively improve quality of life.^9^ Furthermore, in patients with chronic heart failure, when the pacing rate was programmed to 90 b.p.m., the global longitudinal strain and free wall longitudinal strain significantly improved for the right ventricle,^10^ which can independently predict the mortality of patients with chronic heart failure.^11^ We previously implanted a pacemaker in a patient with atrial fibrillation complicated by slow ventricular response (<50 b.p.m.) and pulmonary hypertension, programming the pacing rate to 90 b.p.m. This patient also demonstrated immediate haemodynamic improvement, along with sustained cardiac function and quality of life enhancement throughout the follow-up period.^12^

Based on these findings and our understanding of the basic haemodynamics of the right heart, we hypothesized that for patients with RHF who have normal LVEF, implanting a permanent pacemaker and increasing pacing rate could enhance CO. Cardiac output is determined by end-diastolic volume, ejection fraction, and HR. Therefore, increasing HR can directly enhance pulmonary and systemic CO if the LVEF is normal, improving systemic perfusion, renal function, exercise capacity, and diuresis, while also promoting forward flow from the right atrium and vena cava into the pulmonary circulation, reducing venous pressures and improving congestion symptoms.

A previous study reported that patients who undergo Fontan surgery can remain asymptomatic for decades,^7^ suggesting that left ventricular pump function may be sufficient to sustain both systemic and pulmonary circulation. Since the patient has an LVEF of 69%, left ventricular function is not expected to be compromised by pacing rate acceleration despite the inability to achieve left bundle branch pacing. This conclusion was confirmed by 12-month follow-up echocardiography. Therefore, we recommend considering this therapy for patients with right heart failure and preserved left ventricular function whose symptoms are not adequately controlled by high-dose diuretics.

## Conclusions

Increasing HR can boost CO in patients with refractory RHF, thereby alleviating symptoms and improving quality of life. However, this conclusion requires validation through larger sample sizes and long-term follow-up. We are continuously enrolling patients to conduct further ongoing research.

## Lead author biography


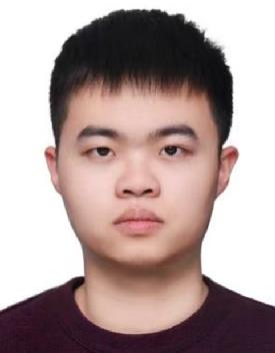


Yanxing Fang is a cardiology intern at Zhongshan Hospital, Fudan University, in Shanghai, China. His research primarily focuses on valvular heart disease, congenital heart disease, and heart failure.

**Consent:** The patient provided informed consent in accordance with the COPE guidelines for this manuscript and associated images.

**Funding:** None declared.

## Data Availability

The data that support the findings of this study are available from the corresponding author upon reasonable request. No restricted data were used in this study.
